# Cost-effectiveness of posaconazole *versus *fluconazole or itraconazole in the prevention of invasive fungal infections among high-risk neutropenic patients in Spain

**DOI:** 10.1186/1471-2334-12-83

**Published:** 2012-04-03

**Authors:** Santiago Grau, Rafael de la Cámara, Francisco J Sabater, Isidro Jarque, Enric Carreras, Miguel A Casado, Miguel A Sanz

**Affiliations:** 1 Hospital del Mar, Barcelona, Spain; 2 Hospital de la Princesa, Madrid, Spain; 3 Health Economics and Outcomes Research IMS Health, Madrid, Spain; 4 Hospital Universitario La Fe, Valencia, Spain; 5 Hospital Clinic, Barcelona, Spain; 6 Pharmacoeconomics & Outcomes Research Iberia, Madrid, Spain

## Abstract

**Background:**

We evaluated the cost-effectiveness of posaconazole compared with standard azole therapy (SAT; fluconazole or itraconazole) for the prevention of invasive fungal infections (IFI) and the reduction of overall mortality in high-risk neutropenic patients with acute myelogenous leukaemia (AML) or myelodysplastic syndromes (MDS). The perspective was that of the Spanish National Health Service (NHS).

**Methods:**

A decision-analytic model, based on a randomised phase III trial, was used to predict IFI avoided, life-years saved (LYS), total costs, and incremental cost-effectiveness ratio (ICER; incremental cost per LYS) over patients' lifetime horizon. Data for the analyses included life expectancy, procedures, and costs associated with IFI and the drugs (in euros at November 2009 values) which were obtained from the published literature and opinions of an expert committee. A probabilistic sensitivity analysis (PAS) was performed.

**Results:**

Posaconazole was associated with fewer IFI (0.05 *versus *0.11), increased LYS (2.52 *versus *2.43), and significantly lower costs excluding costs of the underlying condition (€6,121* versus *€7,928) per patient relative to SAT. There is an 85% probability that posaconazole is a cost-saving strategy compared to SAT and a 97% probability that the ICER for posaconazole relative to SAT is below the cost per LYS threshold of €30,000 currently accepted in Spain.

**Conclusions:**

Posaconazole is a cost-saving prophylactic strategy (lower costs and greater efficacy) compared with fluconazole or itraconazole in high-risk neutropenic patients.

## Background

Patients with neutropenia as a result of chemotherapy for acute myelogenous leukaemia (AML) or myelodysplastic syndrome (MDS) are at high risk of developing invasive fungal infection (IFI) [1-7]. Early diagnosis and treatment of IFI are difficult and, as such, are associated with high mortality rates in neutropenic patients. Hence, prophylaxis of IFI has become a commonly used strategy to reduce overall morbidity-mortality rates in patients with haematologic malignancies [8].

Posaconazole is a new-generation oral azole [9] that has been demonstrated to be superior to standard azole therapy (SAT; fluconazole or itraconazole) in preventing IFI and reducing overall mortality in high-risk neutropenic patients [10]. In a recent study, posaconazol was shown not only to be as efficacious as fluconazole in the prevention of IFI but also superior to fluconazole in the prevention of invasive aspergilosis (proven or probable) in haematopoietic stem cell transplant patients with graft-versus-host disease (GHVD) [11]. As such, posaconazole is recommended in major clinical guidelines [12-15] as prophylaxis for neutropenic patients with AML or MDS (category 1/A-I).

From the National Health Service (NHS) point of view, the financial sustainability of new health interventions is a fundamental priority due to the limitation of the financial resources [16]. Consequently, the authorities responsible for health-care provision require that new therapeutic or preventative alternatives bear information regarding their efficacy, safety, therapeutic usefulness and efficiency (cost-effectiveness) [17]. In this sense, the financial burden of IFI is consistently high, essentially in high-risk neutropenic patients and those who require a protracted stay in the intensive care unit [18-23]. The economic impact of diagnosis and treatment of IFI is related to the cost of the acquisition of the anti-fungal agent, the extra costs of hospitalisation, the cost of diagnostic tests, laboratory analyses and complementary tests, the diagnosis and treatment of the adverse effects of the treatment, the management of the therapeutic failures, and relapses that require the administration of second-line anti-fungal agents [24,25]. Disease burden and high mortality rates oblige health authorities to be aware of efficient anti-fungal prophylaxis in patients at high risk of suffering IFI such as, for example, patients with neutropenia following intensive chemotherapy. However, despite several studies having shown efficacy in specific groups of neutropenic patients at high risk of IFI [26-28], there is a relative dearth of economic evaluations of prophylactic anti-fungal agents.

Posaconazole has been shown to be more efficacious than SAT for the prophylaxis of AML or MDS among high-risk neutropenic patients. However, the cost of the drug is higher than fluconazole and itraconazole. Therefore, the objective of this study was to evaluate the cost-effectiveness of posaconazole compared to SAT for the prevention of IFI in patients with AML or MDS who are at high risk of developing an IFI as a result of chemotherapy-induced neutropenia. The perspective was the Spanish NHS, which is a system of universal health-care provision for 45 million citizens. The data for the present analyses were based on the randomised phase III trial conducted by Cornely et al [10]. The outcomes of the analyses would be useful not only from the Spanish perspective but also for those European countries with a similar health service to that in Spain.

## Methods

A decision analytic model was constructed to evaluate the cost-effectiveness of posaconazole *versus *SAT in preventing IFI in high-risk neutropenic patients. Evidence of clinical effectiveness of both alternatives was based on a randomised study [10] that compared the efficacy and safety of posaconazole with that of SAT as prophylaxis for patients with protracted neutropenia. The trial was conducted following good clinical practice guidelines and the Declaration of Helsinki of the World Medical Association. It was published on clinicaltrials.gov (NCT00044486) and it was approved by the institutional review board or ethics committee at each participating center. The numbers of IFI avoided and the number of life years saved (LYS) with each of the alternative therapies were used to measure the economical benefits. Similarly, we assessed the total cost of treatment including the costs of the prophylactic anti-fungal drug, preparation and administration of posaconazole or SAT, and the treatment of the IFI cases in each group. Based on this information we calculated the incremental cost-effectiveness ratio of the most effective strategy versus the least effective, i.e. the incremental cost for each IFI avoided and the cost for each LYS with posaconazole compared to fluconazole or itraconazole. Model assumptions and parameters of resource use were decided in consultation with an advisory group. The perspective adopted was that of the Spanish NHS, with costs and benefits discounted at an annual rate of 3% after the 1^st ^year of treatment [29].

### Decision-analytical model

Using Microsoft Excel 2003 for Windows^®^, an interactive economic evaluation model was developed to assess the clinical benefits obtained with each therapeutic option, and to estimate the associated health costs of patients receiving prophylaxis with posaconazole or SAT for the prevention of IFI. The costs associated with the prevention or treatment of the IFI that may arise in each of the treatment groups were calculated.

**Figure 1:**
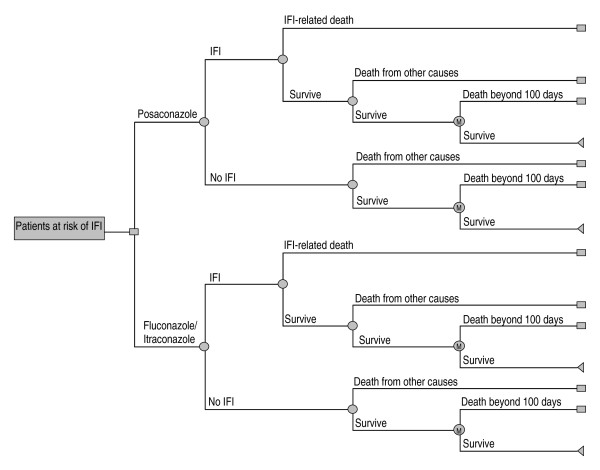
**Decision-tree model of posaconazole* versus *SAT (standard azole treatment; fluconazole or itraconazole) for the prevention of invasive fungal infection (IFI) among high-risk neutropenic patients**. M = Markov model.

The model consists of two integrated parts. The first part is a decision tree (Figure 1) that reproduces, in the 100 days post-treatment, the clinical outcomes for the patients treated with posaconazole or with SAT in a clinical trial comparing both strategies [10]. The decision tree starts with a decision node that describes the prophylaxis selection for IFI, either posaconazole or SAT. After prophylaxis initiation, the patient can develop IFI according to the probabilities described in the clinical trial (Table 1). The model also takes into account the probability of survival or death once the IFI occurrs. The model takes into account not only the patients who do not develop IFI but also those who survive the IFI, as well as the probability of death from other causes not related to the IFI during the initial 100 days of prophylaxis.

**Table 1:** Clinical data parameters used in the model; base-case and sensitivity analysis

Sensitivity analysis						
	**Base-case estimate**		**Deterministic**		**Probabilistic**	

		**Reference**		**Reference**	**Distribution**	**SD**

**Within first 100 days of prophylaxis**						

Probability of an invasive fungal infection (IFI)						

Posaconazole	0.05	Cornely[10]	0.0344 -0.0573	Assumption	Beta	0.0120

SAT	0.11	Cornely[10]	0.0825 -0.1375	Assumption	Beta	0.0181

Probability of an IFI-related death						

Posaconazole	0.36	Cornely[10]	0.2678 -0.4464	Assumption	Beta	0.1247

SAT	0.48	Cornely[10]	0.3636-0.6060	Assumption	Beta	0.0857

Probability of death from other causes (non IFI-related)						

Posaconazole	0.16	Cornely[10]	0.1185-0.1975	Assumption	Beta	0.0148

SAT	0.16	Cornely[10]	0.1185-0.1975	Assumption	Beta	0.0148

**After first 100 days of prophylaxis**						

Relative survival associated with acute myelogenous leukaemia (AML)						

IFI	0.21	NCI[32]	0.16-0.26	Assumption	Gamma	0.000

No IFI	0.21	NCI[32]	0.16-0.26	Assumption	Gamma	0.000

Relative survival associated with myelodysplastic syndrome (MDS)						

IFI	0.08	Kantarjian[33]	0.06-0.10	Assumption	Gamma	0.000

No IFI	0.08	Kantarjian[33]	0.06-0.10	Assumption	Gamma	0.000

*SAT *standard azole treatment (fluconazole 81% patients or itraconazole 19% patients) *SD *standard deviation

The second part of the model consists of an additional Markov model [30], which extrapolates the results of the clinical trial and simulates the progression of the disease over the long-term course of the patients' life, i.e. beyond the first 100 days of prophylaxis. The patients who survive the initial 100 days enter into this Markov model which, following the monthly cycles, projects the risk of death from the underlying disease (AML or MDS) or from whatever cause, independently of whether or not the patients had had an IFI [31]. In this case, the relative survival values obtained from the literature were 0.21 for AML [32] and 0.08 for MDS [33] or other causes (Table 1).

### Clinical data

Clinical efficacy data used in the analyses were collected from a clinical trial that compared posaconazole *versus *fluconazole or itraconazole in the prevention of IFI in high-risk patients with neutropenia [10]. This was a prospective, randomised, multicenter study in which 304 patients were assigned to receive posaconazole and 298 patients SAT (fluconazole in 81% and itraconazole in 19% of patients). The patients received 200 mg posaconazole in oral suspension three times a day (total daily dose: 600 mg), 400 mg fluconazole in oral suspension once a day (total daily dose: 400 mg) or 200 mg itraconazole in oral solution twice a day (total daily dose: 400 mg). Prophylaxis was administered with each chemotherapy cycle, and was continued until recovery from neutropenia and complete remission, or until occurrence of an IFI, or for up to 12 weeks post-randomisation, whichever came first. Patients were followed-up for 100 days post-randomisation, and for 30 days after the last dose of the study drug administered during the last chemotherapy cycle. Proven or probable IFI occurred during the treatment phase in 7 of the 304 patients (2%) in the posaconazole group and in 25 of the 298 patients (8%) in the SAT group; absolute reduction in the posaconazole group of -6% (95%CI: -9.7 to -2.5%; p < 0.001). During the 100-days of the post-randomisation period, 14 of 304 patients (4.6%) in the posaconazole group had a proven or probable fungal infection, compared to 33 of the 298 patients (11%) in the SAT group (p = 0.003) (Table 1). Two patients (1%) in the posaconazole group had invasive aspergillosis *versus *20 (7%) in the SAT group (p < 0.001). The mean (± SD) time to IFI was 41 ± 26 days in the posaconazole group and 25 ± 26 days in the SAT group. Survival was significantly longer among patients treated with posaconazole than among patients in the SAT group. Of the 304 patients in the posaconazole group, 49 (16%) died during the study period, as did 67 of 298 patients (22%) in the SAT group (p = 0.048). The incidence of adverse events was similar among both treatment groups, 52% in the posaconazole group vs 59% in the SAT group [10]. Kaplan-Meier analysis of the all-cause time-to-death at the end of the 100-day period post-randomisation showed a significant survival benefit in favour of posaconazole over fluconazole or itraconazole (p = 0.04). The data on efficacy of both therapeutic options are summarised in Table 1.

### Patient population

The economic evaluation was performed in patients with neutropenia resulting from chemotherapy for AML or MDS and who were at high risk of developing an IFI. The patient population studied correspond to the patients at baseline in the study by Cornely et al [10].

### Cost estimation

To determine the costs and benefits of the treatments under comparison, the perspective used was that of the Spanish NHS and, as such, only the following direct health costs were considered (Table 2).

**Table 2:** Unitary costs* and treatment duration parameters used in the model; base-case and sensitivity analysis

	Base-case estimate		Sensitivity analysis			
			Deterministic		Probabilistic	
		Reference		Reference	Distribution	SD
Total treatment cost per day						
Posaconazole	103.69		77.77 - 129.61	Assum.	Gamma	0.000
Fluconazole	16.93		12.70 - 21.16	Assum.	Gamma	0.000
Itraconazole	21.85		16.39 - 27.31	Assum.	Gamma	0.000
Drug cost per day						
Posaconazole	90.00	CGCOF[34]				
Fluconazole	8.00	CGCOF[34]				
Itraconazole	8.95	CGCOF[34]				
Preparation, administration and monitoring treatment cost per day						
Posaconazole	13.69	Gisbert[35]				
Fluconazole	8.93	Gisbert[35]				
Itraconazole	12.90	Gisbert[35]				
Treatment duration						
Posaconazole	29	Cornely[10]				
Fluconazole	24	Cornely[10]				
Itraconazole	29	Cornely[10]				
Cost of an invasive fungal infection (IFI)						
In-patient cost	67,984	Grau[36]*	50,988 - 84,980	Assum.	Gamma	

*In euros at November 2009 prices

• Costs of the prophylactic treatment used. The cost of the pharmaceutical preparation (exfactory price) was obtained from the medication database of the General Spanish Council of Pharmacists [34]. The model used the values of daily dosage and treatment duration of posaconazole, fluconazole or itraconazole as established in the clinical trial by Cornerly et al [10].

• Cost of preparation, administration and monitoring the anti-fungal treatment. Nurse time cost of 15, 5 and 10 min for the preparation of posaconazole, fluconazole and itraconazole, respectively, and the costs of the three drugs requiring hepatic function monitoring twice a week over the period of prophylaxis. The unit costs of these resources were obtained from the SOIKOS database [35] and updated to values in euros of November 2009.

• Cost of IFI management. The cost per IFI of €67,984 was considered for either treatment option. This value was obtained from the 2005 update of the cost of IFI in an economic evaluation of voriconazol *versus *conventional amphotericin B in the treatment of invasive aspergillosis in Spain [36]. The direct costs of the drug treatment, anti-fungal medication, hospitalisation and laboratory tests were included in this evaluation.

The model did not include the cost of the adverse events nor the cost of management of the underlying disease (AML or MDS) since these costs would be common and similar to both treatment groups. All the costs are expressed in terms of euros at November 2009 values.

### Cost-effectiveness analysis

The time horizon of the study was 100 days, and lifetime of the patient. Hence, for each prophylactic alternative we calculated the number of IFI avoided in 100 days, and the number of LYS over the long-term of the patient's life. Similarly, we obtained the total cost for each treatment group including the costs of the prophylaxis (drug, preparation and administration) and of the treatment of the IFI that may arise. From these results we calculated the incremental cost per IFI avoided, and the incremental costs for each LYS with the more effective treatment (in the present case posaconazole), compared to the less effective SAT (fluconazole or itraconazole).

If the result of the modelling indicated that one of the therapeutic options was more efficacious (less IFI or greater number of LYS) and, in turn, had a lower total cost compared to the other option, this would establish therapeutic dominance. If, as well, a saving is produced relative to the alternative option, then it would be unnecessary to calculate the incremental cost-effectiveness ratio [37]. If, on the other hand, one of the options evaluated was more efficacious but more costly than the alternative, the incremental cost-effective ratio is calculated relative to the less costly alternative. In the analysis we considered that one of the strategies is efficient if the cost per LYS is less than the threshold of efficiency currently accepted in Spain, and which has been established at < €30,000 euros per LYS [38].

### Sensitivity analysis

We performed a deterministic univariate sensitivity analysis with the objective of assessing the robustness of the model, and the consistency of the assumptions used in the model. The parameters modified were those that were most relevant in the model or those with the greatest uncertainty. These were the efficacy of prophylaxis, the risk of death from IFI, and the cost of treatment of the IFI with a value of 75% or 125% of the value used in the base case estimations, except for the probability of experiencing an IFI that a wider range was considered (Tables 1 and 2). Further, a sensitivity analysis was performed applying a discount rate of 0% and 5% to the costs and the outcomes [29].

A multivariate probabilistic sensitivity analysis (PSA) was also performed using 1,000 second-order Monte Carlo simulations. The assumptions of the model were randomly modified based on probability functions described in Tables 1 and 2.

## Results

### Base case analysis

In the base case over the 100 first days of prophylaxis, the IFI per patient in the SAT group was 0.11 versus 0.05 in the posaconazole group (Table 3), i.e. posaconazole avoided a mean of 0.06 IFI per patient.

**Table 3:** Results of the base case of posaconazole* versus* SAT in the prevention of IFI among high-risk neutropenic patients

Strategy	Total costs*	IFI events	LYS	ICER (cost per IFI avoided)	ICER (cost per LYS)
Posaconazole	6,121	0.05	2.52		

SAT	7,928	0.11	2.43		

Difference^†^	-1,807	-0.06	0.09	Dominant^‡^	Dominant^‡^

*In euros at November 2009 prices

^†^Difference between posaconazole and SAT

^‡^Dominant strategy: posaconazole has lower cost and higher efficacy (measured as IFI avoided and LYS) compared to SAT (standard azole treatment: fluconazole 81% patients/itraconazole 19% patients)

In the projection of progression of the disease over the life-term of the patient, the treatment with posaconazole achieved better clinical results, increasing the life expectancy of the patients by 2.52 years, compared to a mean increase of 2.43 years in the SAT group. As such, posaconazole produces an increase of 0.09 in LYS (Table 3).

The total costs of treatment in the SAT group was €7,928 per patient (Table 3), €450 related to the prophylactic drug used in avoiding IFI (drug costs, administration and monitoring) and €7,478 associated with the costs of treatment of FI in the patients with neutropenia. In the group of patients treated with posaconazole, the average cost per patient was €6,121, of which €3,007 was due to the anti-fungal treatment and €3,114 for the management of IFI. The final result was a saving of €1,807 per patient treated with posaconazole compared to the patients who received SAT prophylaxis with fluconazole or itraconazole.

Table 3 summarises the costs and the benefits (IFI avoided and LYS) obtained for each treatment group. The prophylaxis with posaconazole is the dominant strategy compared to prophylaxis with SAT i.e. the clinical outcomes were better and with lower overall cost.

### Deterministic sensitivity analysis

The results of the univariate deterministic sensitivity analysis are summarised in Table 4. All the outcomes are consistent with the base case, i.e. for all the variations of the parameters introduced into the model; posaconazole is the dominant strategy over that of SAT.

**Table 4:** Results of the deterministic sensitivity analysis of posaconazole *versus* SAT in the prevention of IFI among high-risk neutropenic patients

Parameter	Sensitivity analysis value	ICER (cost per IFI avoided)	ICER (cost per LYS)
Probability of IFI; Posaconazole	0.025	Dominant*	Dominant*
	
	0.075	Dominant*	Dominant*

Probability of IFI; SAT	0.075	Dominant*	Dominant*
	
	0.15	Dominant*	Dominant*

Probability of an IFI-related death; Posaconazole	0.2678	Dominant*	Dominant*
	
	0.4464	Dominant*	Dominant*

Probability of an IFI-related death; SAT	0.3636	Dominant*	Dominant*
	
	0.6060	Dominant*	Dominant*

Probability of death from other	0.1185	Dominant*	Dominant*
	
causes; non IFI-related	0.1975	Dominant*	Dominant*

Relative survival; AML	0.16	Dominant*	Dominant*
	
	0.26	Dominant*	Dominant*

Relative survival; MDS	0.06	Dominant*	Dominant*
	
	0.10	Dominant*	Dominant*

Total treatment cost per day; Posaconazole	77.77	Dominant*	Dominant*
	
	129.61	Dominant*	Dominant*

Total treatment cost per day; Fluconazole	12.70	Dominant*	Dominant*
	
	21.16	Dominant*	Dominant*

Total treatment cost† per day; Itraconazole	16.39	Dominant*	Dominant*
	
	27.31	Dominant*	Dominant*

Discount rate for costs and benefits	0%	Dominant*	Dominant*
	
	5%	Dominant*	Dominant*

*Dominant strategy: posaconazole has lower cost and higher efficacy (measured as IFI avoided and LYS) compared to SAT (standard azole treatment: fluconazole/itraconazole)

^†^In euros at November 2009 prices

### Probabilistic sensitivity analysis

**Figure 2:**
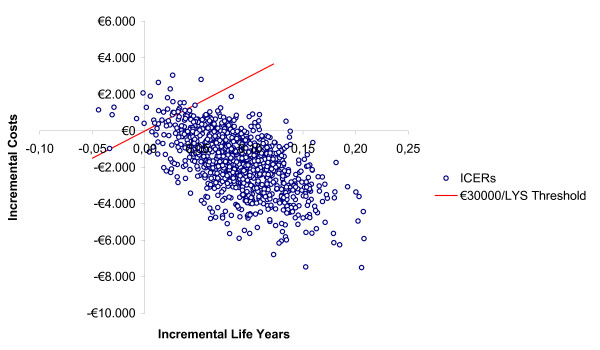
**Probabilistic sensitivity analysis (PSA); incremental cost-effectiveness ratio (ICER) of posaconazole *versus *SAT (standard azole treatment; fluconazole or itraconazole) in the prevention of invasive fungal infection (IFI) among high-risk neutropenic patients (ICER threshold considered is €30,000 per life-year saved)**.

**Figure 3:**
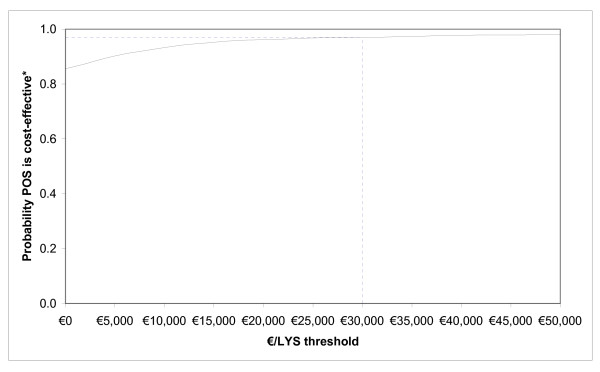
**Cost-effectiveness acceptability curve; probability that posaconazole is cost-effective relative to SAT (standard azole treatment; fluconazole or itraconazole)**.

The results of the PSA show that there is a probability of 85% that posaconazole is a cost-saving strategy, compared to SAT (Figure 2) and a probability of 97% that the incremental cost-effectiveness ratio for posaconazole *versus *SAT is below the estimated €30,000 per LYS threshold currently accepted in Spain (Figure 3).

A final sensitivity analysis was performed, to find at what values results changed. Only when the probability of experiencing an IFI was identical in both groups, SAT was dominant over posaconazole treatment due to lower treatment costs. The treatment with posaconazole would not be more cost-effective if the incremental benefit over SAT was reduced from 6% to 2%, as the ICER would be higher than €30,000.

## Discussion

The findings from this study show that, from the perspective of the Spanish NHS, posaconazole is more effective than standard azoles (in the present case fluconazole or itraconazole) in preventing proven or probable IFI. The outcome is a reduction in overall mortality and a longer IFI-free survival among high-risk neutropenic patients with AML or MDS. Under most conditions in the model, posaconazole is the dominant strategy compared to SAT, i.e. patients who are treated with posaconazole have avoided a greater number of IFI with a higher survival rate while, at the same time, have lower total costs compared to patients receiving SAT. Deterministic sensitivity analyses showed that the modification of key parameters of the model had very little impact on the cost-effectiveness of posaconazole and, as such, the model is considered consistent in all the established assumptions.

Some limitations of the study need to be taken into account when interpreting these results. Firstly, the data on efficacy of the two therapeutic alternatives evaluated were obtained from a single clinical trial [10] so the differences in frequency and distribution of fungal species in real clinical setting could affect the efficacy results reported. Also, the data on resource use in the IFI were estimated from the published literature and, in a retrospective manner, by a panel of experts (authors of this manuscript). However, a probabilistic sensitivity analysis was conducted to evaluate the level of uncertainty associated with the inputs and assumptions of the model and, as well, to determine the interactions between the variables analysed. This analysis showed that there is an 85% probability that posaconazole is a cost-saving strategy compared to SAT and that the probability that the incremental cost-effectiveness ratio for posaconazole *versus *SAT is below the accepted threshold in Spain (currently €30,000 per LYS) is 98%. However, with regards to external validity required by decision makers, it would be of considerable interest to evaluate whether these results are transferable to other settings. There may be further doubts regarding the benefits encountered in the clinical trial (the data from which were used in the present model) and whether these outcomes would apply to patients treated in routine clinical practice. On the other hand, the quality-of-life related to health in the outcomes of the patients treated with posaconazole or SAT was not taken into account, nor was the cost of the management of adverse events. However, in the referenced clinical trial [10], the incidence of adverse events was similar in both treatment groups. Similarly, the costs of management of the underlying diseases were not incorporated in the model since these were considered to be equal in the two alternative treatments and, consequently, would not have an impact on the final outcomes of the analyses.

Although our results are only valid for the Spanish National Health Care System due to the local nature of costs, our findings are in line with previous cost-effectiveness analyses of posaconazole in the prevention of IFI among high-risk neutropenic patients with AML or MDS, based on Cornely study [10], conducted in other European countries and in the USA. All the cost-effectivenes analyses followed similar methodology and show that the incremental cost per LYS of posaconazole *versus *SAT is below the threshold commonly accepted in each of the countries as ceiling threshold for determining society's willingness to pay for a treatment, even when it is a dominant strategy (cost-saving) [28,39-43].

## Conclusion

Our results suggest that prophylactic posaconazole in neutropenic patients with acute myelogenous leukaemia or myelodysplastic syndromes could potentially increase patient life expectancy and reduce the overall health-care budget.

## Competing interests

R. de la Cámara, E. Carreras, M.A. Sanz and I. Jarque: have received honoraria for speaking at symposia organised on behalf of Pfizer, Merck Sharp & Dohme (MSD), Schering-Plough and Gilead Science and has sat on advisory boards for antifungal agents on behalf of MSD, Schering-Plough, Pfizer and Gilead; S. Grau: has received honoraria for speaking at symposia organised on behalf of Pfizer; M.A. Casado has served as an external consultant for Schering-Plough S.A; F.J. Sabater was an employee of Schering-Plough at the moment of manuscript first submission.

## Authors' contributions

SG, RC, MAS, EC and IJ participated in data interpretation and writing the manuscript. MAC participated in study concept, designing the study, data interpretation and writing the manuscript. FJS participated in study concept. All the authors read and approved the final manuscript.

## Pre-publication history

The pre-publication history for this paper can be accessed here:

http://www.biomedcentral.com/1471-2334/12/83/prepub
